# Suspected autoimmune-mediated dissociative symptoms

**DOI:** 10.1038/s41380-025-02926-0

**Published:** 2025-02-26

**Authors:** Dominique Endres, Elena Reinhold, Christian Klesse, Katharina Domschke, Harald Prüss, Ludger Tebartz van Elst

**Affiliations:** 1 Department of Psychiatry and Psychotherapy, Medical Center - University of Freiburg, Faculty of Medicine, University of Freiburg, Freiburg, Germany; 2 German Center for Mental Health (DZPG), Partner Site Berlin, Berlin, Germany; 3 Department of Neurology and Experimental Neurology, Charité - Universitätsmedizin Berlin, Berlin, Germany; 4 German Center for Neurodegenerative Diseases (DZNE) Berlin, Berlin, Germany

**Keywords:** Neuroscience, Diagnostic markers

## Introduction

Dissociative disorders are usually interpreted as psychoreactive sequelae of stressful life events or conflicts that patients may avoid through symptoms [1]. Dissociative symptoms can occur in other mental disorders, such as borderline personality disorder, learning disability, autism spectrum disorder, or schizophrenia. Little is known about secondary forms and their underlying biological mechanisms. Therefore, this paradigmatic case study of a patient with predominant dissociative symptoms and associated novel anti-central nervous system (CNS) antibodies in the cerebrospinal fluid (CSF) is presented.

## Case study

Now in her mid-thirties, the patient has had a dissociative syndrome since adolescence. In the last two years, the symptoms worsened to the extent that she consistently lost touch with reality several hours in a day, which resulted in her being absent and frozen. Sometimes, especially when she was alone she could switch to a children’s voice, speaking with and defending herself against an imaginary other person. In such situations she also occasionally engaged in auto-aggressive behavior in hitting herself. Previously assigned diagnoses based on the patient’s symptomatology included depersonalization disorder, dissociative movement disorder, dissociative identity disorder, dissociative trance states, cognitive dysfunction, affective disorder, and schizophrenia. Because of intermittent reports of hearing her thoughts in sensory overload situations and the symptoms described above, the diagnosis of paranoid schizophrenia was currently discussed as a relevant differential diagnosis, however, it was felt that the persistent dissociative syndrome was clearly dominating the overall clinical impression. There was no evidence for an underlying learning disability (she reached high school graduation “Abitur” in Germany). Various pharmacological attempts either had no positive effects (neuroleptics, antidepressants, mood stabilizers, or naltrexone) or were not tolerated (e.g., Stevens–Johnson syndrome under lamotrigine). Electroconvulsive therapy provided only transient improvement in the patient’s history. The patient took benzodiazepines regularly.

**Fig. 1:**
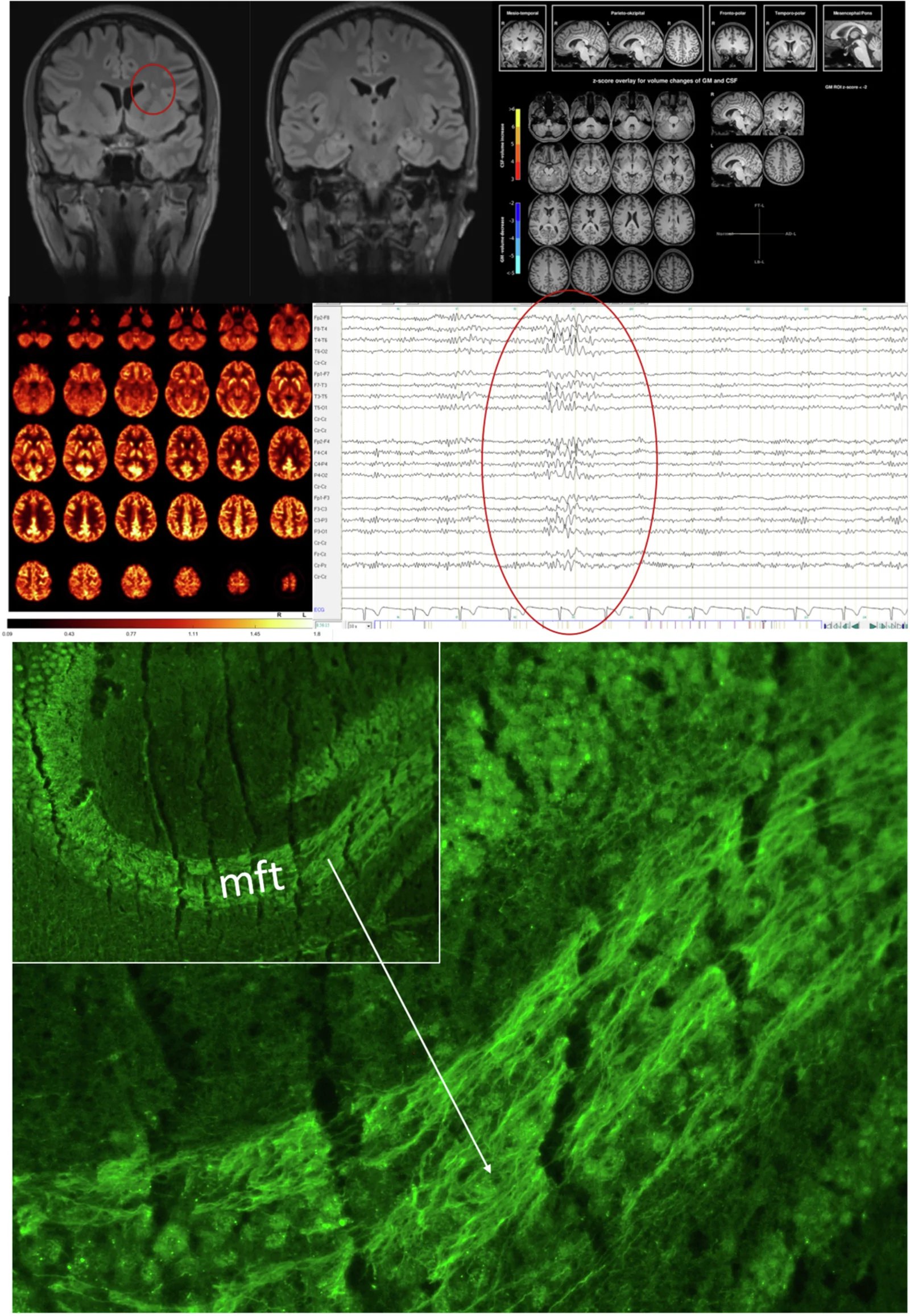
Magnetic resonance imaging (MRI) revealed only non-specific white matter lesions (see exemplary within red circle). Veobrain analysis of the MRI showed no volume loss (https://www.veobrain.com/?page=veomorph). [^18^F]fluorodeoxyglucose positron emission tomography (FDG-PET) of the brain showed normal findings. Whole-body FDG-PET (not shown) revealed no evidence of tumor. Electroencephalography (EEG) showed intermittent slowing (see exemplary red marker in EEG). Tissue-based assays using cerebrospinal fluid (CSF) identified strong immunoglobulin (Ig) G antibody binding to the mossy fiber tract (mft) in the hippocampus. The well-characterized anti-neuronal and anti-glial autoantibodies were negative (not shown here). Serum was tested negative for paraneoplastic IgG antibodies against intracellular antigens (Yo, Hu, CV2/CRMP5, Ri, Ma1, Ma2, SOX1, Tr/DNER, Zic4, GAD65, amphiphysin) and glial antigens (MOG/AQP4). Neuronal IgG cell surface antibodies (NMDA-R, LGI1, CASPR2, GABA-B-R, AMPA1-R, AMPA2-R, DPPX) were negative in serum and CSF. The routine CSF analyses (not shown here) detected intrathecal synthesis for all three Ig isotypes (IgG, IgM, and IgA) and oligoclonal bands in CSF. Both the white blood cell count (4/µL; reference: <5/µL) in the CSF and the albumin quotient (2.9; reference: <6.3) were normal.

The diagnostic work-up was based on established protocols [2, 3]. Broad laboratory analyses did not reveal relevant findings. Electroencephalography (EEG) showed intermittent slowing. An independent component analysis of the EEG identified spindle-shaped bursts of waves around 10 Hz superposing on a slow potential, mainly in the left temporal and occipital lobe. Magnetic resonance imaging (MRI) showed non-specific white matter changes, and automated morphometry identified no volume loss (https://www.veobrain.com/?page=veomorph). The [^18^F]fluorodeoxyglucose positron emission tomography (FDG-PET) of the brain was normal, and the FDG-PET of the whole body detected no evidence of malignancy. A cerebrospinal fluid (CSF) analysis revealed an inflammatory constellation with intrathecal three-class immunoglobulin (Ig) synthesis (IgG 64%, IgM 17%, IgA 32%; reference <10%) and CSF-specific oligoclonal bands (OCBs). The well-characterized anti-CNS antibodies against cell surface, intracellular, and glial antigens remained negative [4]. In tissue-based assays on unfixed mouse brain sections [5], moderate ubiquitous myelin binding and very strong IgG binding against the hippocampal mossy fiber tract in serum and CSF were detected (Fig. 1 and Supplementary Table 1).

Immunotherapeutic approaches included a high-dose steroid pulse twice (1000 mg methylprednisolone over five days with oral tapering) and a plasmapheresis cycle (over five days) once. After the first treatment with steroids and plasmapheresis, there was a relevant but not long-lasting reduction in dissociations. A repeated steroid pulse treatment six months later resulted in only minimal improvement. Further immunotherapy was not desired by the patient or her parents.

## Discussion

The presented patient mainly suffered from predominant severe and persistent dissociative symptoms. The CSF analysis revealed clear signs of chronic neuroinflammation with intrathecal three-class synthesis and OCBs. In addition, anti-CNS antibodies with strong binding to the mossy fiber tract of the hippocampus were identified. In this constellation, an autoimmune-mediated CNS process was suspected.

A PubMed search for the identification of similar cases (on 25 August 2024 with the search strategy “dissociative disorder AND autoimmune”) resulted in only 23 hits. Among these were two case reports of anti-NMDA-R encephalitis and stiff-person syndrome initially misdiagnosed as a dissociative disorder [6–8]. One case, each with underlying Graves’ hyperthyroidism, with acute disseminated encephalomyelitis (ADEM) and systemic lupus erythematous, was also found [9–11]. All of these diagnoses were largely excluded in our patient.

Since the well-characterized anti-CNS antibodies in serum and CSF remained negative but specific and strong IgG-binding to the entire mossy fiber tract was identified in the tissue-based assay, a novel CNS antigen can be assumed. Taking into additional consideration the inflammatory CSF signals, the EEG alterations, and the previous therapy resistance, immunotherapeutic attempts were made with high-dose steroids and plasmapheresis, which led only to an intermediate but not a sustained clinical improvement. Whether further escalation of immunotherapy could still lead to an improvement in such constellations with a long course of disease will have to be studied in future research.

The antibodies bound strongly and specifically to the mossy fiber tract in the hippocampus. The mossy fibers represent the axons of granule cells that form synapses with the interneurons and pyramidal cells of the hippocampus. The peculiarity of granule cells is that glutamatergic and GABAergic phenotypes exist, which allows glutamatergic (excitatory) and GABAergic (inhibitory) responses to occur in mossy fibers and postsynaptic cells. This normally succeeds in maintaining the excitation–inhibition balance [12]. The detected antibodies may have altered the balance in terms of over-excitation similar to ketamine overdose [13]. In principle, this may also explain the high need for benzodiazepines in the patient.

As a limiting factor, it must be mentioned that the target antigen and the functionality of the antibodies are still unclear. Identification of the underlying antigen, such as by use of immunoprecipitation and mass spectrometry, may foster the understanding of underlying pathomechanisms [14]. Due to the suspected secondary cause, the complex clinical symptoms in the presented patient (diagnosed with several different mental illnesses earlier) were described on a syndromal level. Additional (semi)-structural interviews were not performed. In clinical practice, a primary paranoid schizophrenia may have been diagnosed. It therefore remains unclear whether the conclusions of this case study are transferable to classical dissociative disorders.

In summary, the case shows that dissociative symptoms may sometimes have autoimmune causes or contribution. A multimodal diagnostic work-up, including CSF analysis and screening for novel anti-CNS antibodies, could be necessary to detect such cases [15].

## Supplementary information

Supplemental Table 1

## Data Availability

All necessary data can be found in the paper.
