# Supplementary material for: Suspected autoimmune-mediated dissociative symptoms
Source: Mol Psychiatry. 2025 Feb 26;30(5):2260–3. doi: 10.1038/s41380-025-02926-0 (PMC12015169; doi:10.1038/s41380-025-02926-0)
Supplement: Supplementary file 1 — Supplemental Table 1 [file 41380_2025_2926_MOESM1_ESM.pdf]

SUPPLEMENTAL TABLE

**Serum antibodies and immunological markers**	
**ANAs (*on HEp-2 cells*), ANCAs (*on EthOH-/formalin-fixed neutrophils*), APAs**	Negative
Complement factors (C3, C4, CH50)	Normal
IgG, IgM and IgA levels	Normal
Immunofixation	Trace evidence of a ***monoclonal gammopathy of the type IgG lambda*** (band very blurred and not typically monoclonal).
CRP	< 3 mg/l (normal)
**Serum pathogens**	
Serology for Lyme disease, lues, CMV, EBV, FSME, HBV, HCV, HIV, toxoplasmosis, tuberculosis, Bartonella henselae	***IgG antibodies against Borrelia clearly positiv***e, IgM antibodies negative. Findings indicate a past Borrelia infection. ***CMV IgG detectable*** (IgM negative). Findings indicate a past CMV infection. ***EBV seropositivity*** is present (past infection). All other serologies were unremarkable.
**Serum anti-neuronal/glial autoantibodies**	
Paraneoplastic IgG antibodies against intracellular antigens (*Yo, Hu, CV2/CRMP5, Ri, Ma1, Ma2, SOX1, Tr/DNER, Zic4, GAD65, amphiphysin*)	Negative
Well-characterized neuronal IgG cell surface antibodies (*NMDA-R, LGI1, CASPR2, GABA-B-R, AMPA1-R, AMPA2-R, DPPX*)	Negative
Anti-MOG/AQP4-IgG antibodies	Negative
Tissue based assay on unfixed murine brain tissue (Prof. Prüss, Charité Berlin, Germany)	***Moderate ubiquitous myelin binding, strongly positive binding on the hippocampal mossy fiber tract (+++).***
**Cerebrospinal fluid**	
White blood cell count	4/µL (ref.: < 5/µL)
Protein concentration	240 mg/L (ref.: < 450 mg/L)
Albumin quotient	2.9 (ref.: < 6.3)
IgG-index	***1.73*** (ref.: < 0.7)
Oligoclonal bands in serum/CSF	***CSF specific oligoclonal bands***
Local IgG/IgA/IgM synthesis	***IgG (64%), IgM (17%), IgA (32%)***
Lyme disease antibody index	IgG antibody index negative (0.6, reference < 1.5). No evidence of intrathecal antibody synthesis.
Well-characterized neuronal IgG cell surface antibodies (*NMDA-R, LGI1, CASPR2, GABA-B-R, AMPA1-R, AMPA2-R, DPPX*)	Negative
Tissue based assay on unfixed murine brain tissue (Prof. Prüss, Charité Berlin, Germany)	***Moderate ubiquitous myelin binding, strongly positive binding on the hippocampal mossy fiber tract (+++).***

**MRI of the neurocranium**<br>Visual assessment<br><br>Automated morphometry	 ***Non-specific FLAIR hyperintensities in the left-hemispheric white matter***.<br>Normal. No atrophic changes.
**EEG**<br>Visual assessment<br>ICA analysis	 ***Intermittent slowing.***<br>***Alpha rhythm at 9, Mu (motor system) at 26.5Hz. Independently, spindle-shaped bursts of waves around 10Hz appear, on a slow potential (delta). Mainly left temporal and occipital, later right also involved. Frequency is quite low - 0.5 per minute, while striking IRDA frequency was detected otherwise from about 7 per minute. Slightly increased frequency after HV.***
**FDG-PET**<br>Brain<br><br><br>Whole body	 Age-appropriate cerebral glucose utilization, in particular no typical pattern for a neurodegenerative or inflammatory brain disease.<br>No evidence of malignancy-suspect hypermetabolic lesions.
**Test of attentional performances**	Average results for psychomotor speed in basal low-requirement stimulus-response tasks in the computer-based examination of concentration and attention. Divided attention and flexibility are unremarkable, ***deficits show up in working memory***. There are also indications of ***slightly increased irritability/distractibility.***

Supplemental Table 1: Diagnostic findings at baseline. Some of the diagnostic investigations were performed more than once, for example, two lumbar punctures were made. In these cases, the most conspicuous finding was noted here. *Abbreviations: ANAs, antinuclear antibodies; ANCAs, anti-neutrophil cytoplasmic antibodies; APAs, antiphospholipid antibodies; AQP4, aquaporin-4; CMV, cytomegalovirus; CRP, C-reactive protein; CSF, cerebrospinal fluid; EBV, Epstein-Barr virus; EEG, electroencephalography; FDG-PET, fluorodeoxyglucose-positron emission tomography; FSME, early summer meningoencephalitis, HAV, hepatitis A virus; HBV, hepatitis B virus; HCV, hepatitis C virus; HIV, Human Immunodeficiency Virus; ICA, independent component analysis; IgA/G/M, immunoglobulin A/M/G; MOG, myelin oligodendrocyte glycoprotein; MRI, magnetic resonance imaging; VZV, varicella zoster virus; WBC, white blood cell.*
